# Low Temperature-Dependent Salmonid Alphavirus Glycoprotein Processing and Recombinant Virus-Like Particle Formation

**DOI:** 10.1371/journal.pone.0025816

**Published:** 2011-10-03

**Authors:** Stefan W. Metz, Femke Feenstra, Stephane Villoing, Marielle C. van Hulten, Jan W. van Lent, Joseph Koumans, Just M. Vlak, Gorben P. Pijlman

**Affiliations:** 1 Laboratory of Virology, Wageningen University, Wageningen, The Netherlands; 2 Intervet Norbio, Bergen, Norway; 3 Intervet International BV, Boxmeer, The Netherlands; University of Texas Medical Branch, United States of America

## Abstract

Pancreas disease (PD) and sleeping disease (SD) are important viral scourges in aquaculture of Atlantic salmon and rainbow trout. The etiological agent of PD and SD is salmonid alphavirus (SAV), an unusual member of the *Togaviridae* (genus *Alphavirus*). SAV replicates at lower temperatures in fish. Outbreaks of SAV are associated with large economic losses of ∼17 to 50 million $/year. Current control strategies rely on vaccination with inactivated virus formulations that are cumbersome to obtain and have intrinsic safety risks. In this research we were able to obtain non-infectious virus-like particles (VLPs) of SAV via expression of recombinant baculoviruses encoding SAV capsid protein and two major immunodominant viral glycoproteins, E1 and E2 in *Spodoptera frugiperda Sf*9 insect cells. However, this was only achieved when a temperature shift from 27°C to lower temperatures was applied. At 27°C, precursor E2 (PE2) was misfolded and not processed by host furin into mature E2. Hence, E2 was detected neither on the surface of infected cells nor as VLPs in the culture fluid. However, when temperatures during protein expression were lowered, PE2 was processed into mature E2 in a temperature-dependent manner and VLPs were abundantly produced. So, temperature shift-down during synthesis is a prerequisite for correct SAV glycoprotein processing and recombinant VLP production.

## Introduction

Global fish-aquaculture has grown extensively over the past 50 years, producing yearly up to 52.5 million tons worth of an estimated US$98.5 billion. Approximately 50% of the world's fish supply is derived from fish farming, of which salmon and trout represent high value cultivates with an output of over 1.5 million tons worth US$7.2 billion per year [1]. Unlike other reared and farmed animals, fish are often cultured in open systems, exposing them to a wide variety of naturally occurring pathogens, in particular fish infecting viruses [2].

Salmonid alphavirus (SAV) is a serious pathogen for European farmed Atlantic salmon, Salmo salar and rainbow trout, Oncorhynchus mykiss and is the etiological agent of pancreas disease (PD) and sleeping disease (SD), respectively. PD is associated with lack of appetite, lethargy, pancreatic and heart lesions and increased mortality up to 48% of infected fish [3]. SD is characteristically presented by fish lying on their side on the bottom of the culture tank due to severe skeletal muscle necrosis [4]. SAV is an unusual alphavirus and differs from other alphaviruses like Sindbis virus (SINV), Semliki forest virus (SFV) or Chikungunya virus (CHIKV) as it solely replicates in fish (mainly salmonids) and has no known arthropod vector. Although SAV has successfully been detected in parasitic salmon louse species, the supposition of lice being a vector for SAV transmission remains to be proven, since active SAV replication within lice has not yet been confirmed [5], [6]. In addition, it has been shown that SAV can be transmitted from fish to fish in cohabitation experiments in the absence of an arthropod vector [7] [8].

SAV belongs to the genus Alphavirus within the Togaviridae family and represents at least six closely related subtypes, of which three are of special interest: salmon pancreas disease virus (SPDV or SAV1) from Ireland, sleeping disease virus (SDV or SAV2) from France and the Norwegian salmonid alphavirus (NSAV or SAV3) [3]. SAV virions are enveloped spherical (∼65 nm) particles and contain a positive sense, single-stranded RNA genome of approximately 12kb [9]. The viral RNA encodes two open reading frames (ORF); the non-structural ORF, which is directly translated from the genomic RNA, and the structural ORF, which is encoded by a 26S sub-genomic mRNA and is processed into 5 structural proteins – capsid, E3, E2, 6K, and E1. SAV transmembrane glycoproteins E1 and E2 are exposed on the virion surface as trimeric spikes, facilitating cell receptor recognition (presumably E2), cell entry via pH-dependent endocytosis (presumably E1) and support budding. E2 also serves an important role in regulating the fusion activity of E1 so that this does not occur before endocytosis [10]. The acidic endosomal environment dissociates the trimeric spike and causes E1 to initiate fusion with the endosomal membrane [11], [12], thereby releasing the nucleocapsid and subsequently the viral RNA into the cytoplasm of the cell.

During translation of the structural polyprotein, the capsid protein is autocatalytically cleaved off from the structural polyprotein to encapsidate newly synthesized genomic RNA. The remaining envelope cassette (E3-E2-6K-E1) is subsequently translocated to the endoplasmic reticulum (ER) and is processed by host signalases at the N-terminal and C-terminal end of 6K, yielding E3E2 (precursor E2; PE2), 6K and E1[13]. The membrane anchored PE2 and E1 form heterodimers and three PE2-E1 dimers will eventually assemble into heterotrimers in the rough ER of infected cells [14], [15], [16], [17]. The presence of E3 within the heterotrimers offers resistance against the acidic environment of the Golgi apparatus, to avoid premature trimer activation by homotrimerization of E1 [13]. In the trans-Golgi system, PE2 undergoes furin-dependent maturation, thereby releasing E3 from E2. However, E3 may remain associated with the E1-E2 trimers in the acidic compartments of the cell and dissociates once the trimers reach the cell surface to prime the spikes for acidic activation [18].

SAV infection in farmed salmonids can be minimized by reducing stress and good hygienic culture methods, such as sea-lice control and proper boat and transporter cleaning and disinfection. Next to these control measures, vaccination in fish has shown to be effective in protecting farmed salmon and trout from SAV infections. Few studies have focused on SAV-specific immune responses in fish, but it has been shown that infected salmonids generate a short-lasting (∼9 months) protective immune response against subsequent SAV infections [19] and that, similar to other alphaviruses, immune cells are involved in dissemination of infection in the host [20], [21]. Although the immune response against SAV is not fully understood, it has been demonstrated in multiple studies that salmonids can be protected against SAV challenge by immunization with inactivated virus formulations and that immunized fish elicit neutralizing antibodies, resulting in viral clearance [8], [22]. Several vaccination strategies have been developed and tested, such as formalin-inactivated viral vaccines, recombinant and attenuated live vaccines [3], [23], [24], [25], [26]. Although these vaccines provide cross-protection to all SAV-subtypes, safety issues such as incomplete inactivation of active virus remain problematic. The use of a subunit vaccine may serve as an elegant alternative for inactivated or live-attenuated vaccines, in particular the use of virus-like particles (VLPs). VLPs are non-pathogenic virus look-alikes, since they are morphologically similar to virions but are non-infectious as they do not contain viral RNA. Recent studies have shown that the expression of the CHIKV-structural polyprotein in culture cells results in the formation of VLPs, which induced a protective immune response in non-human primates [27]. However, the low temperature replication and assembly of SAV are potentially major hurdles to the production of SAV VLPs.

This study focuses on the genesis of SAV VLPs using the recombinant baculovirus-insect cell expression system. This expression system has proven to be an efficient and safe way to express heterologous proteins on large scale in insect cells, which resulted in the production of several commercially available human and veterinary viral vaccines [28], [29], [30], [31], [32]. The recombinant insect cell expression system is based upon the exchange of the baculovirus polyhedrin gene for the heterologous gene of interest. Polyhedrin expression is controlled by the strong polyhedrin promoter, thereby enabling high heterologous protein expression. Next to high level protein expression, insect cells enable post-translational protein modification and accurate folding [29], [33] and several studies have shown efficient alphavirus protein expression, using recombinant baculoviruses [34], [35], [36]. In addition, the baculovirus-insect cell expression has been shown to be an elegant method for VLP production of enveloped and non-enveloped arboviruses [37]. In this study, we show that the formation of SAV VLPs in insect cells does not take place under standard conditions, but that it is dependent on the level of processing of the envelope glycoprotein E2. In addition, we show that E2 processing is a function of temperature and is only complete at low temperatures. Finally, we present an optimized production process involving a temperature-shift regime to allow the efficient secretion of SAV VLPs in the culture fluid of baculovirus-infected insect cells.

## Materials and Methods

### Cells and viruses

Adherent *Spodoptera frugiperda* (*Sf*9*)* cells (Invitrogen) were maintained as monolayer cultures in Sf-900 II medium (Gibco), supplemented with 5% fetal calf serum. Recombinant baculoviruses were generated using a modified *Autographa californica* multicapsid nucleopolyhedrovirus (AcMNPV) backbone. Promoter sequences and large parts of the coding sequences of cathepsin and chitinase were deleted from the bacmid backbone [38]. The resulting AcΔcc was further modified by deletion of the p10 promoter and ORF. These elements were replaced by a zeocin resistance marker by Lambda Red recombination [39], [40]. Modified LoxP sequences flanking the zeocin resistance marker were used for subsequent removal of the resistance marker by Cre recombinase [41]. The pFastBac1/SAV3 was constructed by cloning the SAV3 structural polyprotein (Genbank accession # AY604235) as a 3957 nt *Eco*RI-*Xba*I fragment into pFastBAC1 (Invitrogen). The recombinant baculovirus AcΔccΔp10 expressing the structural cassette of SAV3 was generated using the Bac-to-Bac baculovirus expression system (Invitrogen), resulting in Ac-SAV3. Recombinant baculovirus titers expressed as tissue culture infectious dose 50 (TCID_50_) per ml, were determined by end point dilution [42]. All infections were performed in serum-free Sf-900 II medium.

### Recombinant baculovirus infections and temperature-shift assay

To analyze protein expression and processing, 1.5×10^6^ *Sf*9-cells were seeded in a 6-wells culture plate and infected with Ac-SAV3 at a multiplicity of infection (MOI) of 10 TCID_50_ units per cell. Infections were performed at 27°C on a shaking platform. Subsequent incubations were performed at 12°C, 15°C, 18°C or 27°C for 14, 10, 7 and 3 days, respectively. The medium fraction was removed and cells were harvested, washed once in phosphate buffered saline (PBS), resuspended in 200 µl PBS and stored at −20°C. To analyze the effect of a temperature-shift on protein processing, cells were infected and harvested as previously described, but were initially incubated at 27°C. Next, cells were transferred to 12°C, 15°C or 18°C at 24hpi, 48hpi or 72hpi.

### Protein analysis

Secreted protein fractions were precipitated by 7% (w/v) NaCl, 2.3% (w/v) polyethylene glycol (PEG)-6000 precipitation. Pellets were resuspended in 100 µl PBS and stored at -20°C. Whole cell lysates and medium fractions were analyzed by sodium dodecyl sulphate polyacrylamide gel electrophoresis (SDS-PAGE) and Coomassie brilliant blue (CBB) staining. Denatured proteins were transferred to an Immobilon membrane (Millipore) and examined by western analysis. Membranes were blocked with 3% skim milk in PBS-0.1% Tween-60 (PBST) for 1 h at room temperature (RT). Membranes were washed in PBST and incubated for 1h at RT with 1∶2000 diluted primary monoclonal antibody (mab) α-E2 17H23 (SAV3) [43] and α-E1 (raised against the N-terminus of SAV3 E1, aa 1-26) in PBST, supplemented with 0.2% skim milk. Membranes were washed 3 times with PBST and incubated for 45 minutes at RT with 1∶3000 diluted Alkaline Phosphatase (AP) conjugated secondary antibody, in PBST supplemented with 0.2% skim milk. Membranes were washed three times in PBST and incubated with AP-buffer for 10 min. Proteins were detected by NBT/BCIP staining (Roche). Antigenic mass was determined by a sandwich immune-capture ELISA using the monoclonal α-E2 17H23 (SAV3) antibody [43].

### PNGase F treatment

Protein fractions were treated with PNGase F (New England Biolabs) to analyze the glycosylation status of SAV3-E2. Cell fractions and the precipitated medium fraction were treated with 1 µl glycoprotein denaturing buffer in 9 µl MilliQ for 10 min at 100°C. The denatured protein-mix was incubated for 1 h at 37°C with 2 µl 10x G7 reaction buffer, 2 µl 10% NP40, 1.5 µl PNGase F and 4.5 µl MilliQ. Treated proteins fractions were analyzed with SDS-PAGE, western blot (WB) and CBB.

### Immunofluorescence assay

*Sf*9-cells were seeded on glass coverslips in 24-wells plate, infected and incubated at 12°C, 15°C, 18°C and 27°C. Cells were fixed with 4% paraformaldehyde in PBS for 5 min at RT, washed with PBS and subsequently incubated with PBS containing 1∶2000 diluted primary monoclonal mouse α-E2 antibody [43] for 1 h at RT. Cells were washed carefully with PBS and treated with 1∶1000 diluted goat α-mouse polyclonal Alexa 546 (Invitrogen) for 1 h at RT in dark conditions. Next, cells were washed and coverslips were fixed on glass slides with a drop of antifade fluoromount-G (Southern Biotech). Cells were analyzed by laser confocal microscopy on a Zeiss LSM 510 Meta, Axiovert 100 m.

### Electron microscopy analysis

Copper 400 square mesh grids (Veco) were hydrophilized by Argon gas discharge. Next, 10 µl sample was applied for 2 min and the excess liquid carefully removed. The grid was washed 5x with MilliQ and stained with 2% uranyl acetate for 20 s. Excess dye was removed, grids were air dried and analyzed with a JEOL JEM 2100 transmission electron microscope. Analysis of fixed cell samples was performed as described [44].

## Results

### Expression of SAV3 structural cassette in *Sf*9 insect cells by recombinant baculoviruses

**Figure 1:**
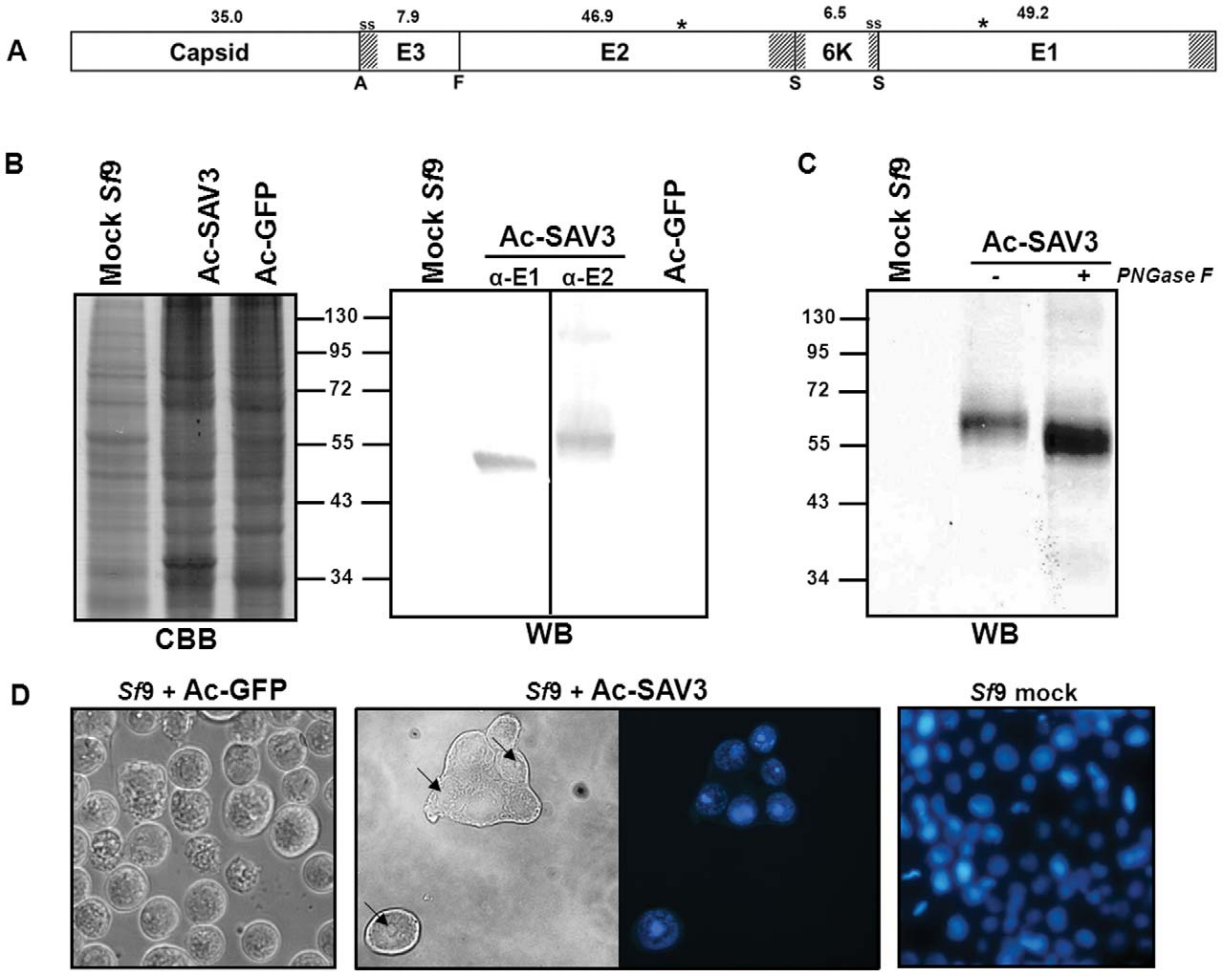
SAV3 structural cassette expression, using recombinant baculoviruses. A) Schematic representation of the SAV3 structural cassette as it is expressed by recombinant baculoviruses. The molecular mass of the proteins are indicated and shaded areas represent transmembrane domains or signal sequences (ss). Autocatalytic (A), furin (F) and signalase (S) cleavage sites are indicated, asterisks represent N-linked glycosylation sites. B) Protein expression in *Sf*9 cells was analyzed by CBB and WB using SAV α-E1 and SAV α-E2 mabs. C) Whole cell lysates were treated with/without PNGase F and analyzed with SAV α-E2 mabs. D) Cells infected with Ac-GFP and Ac-SAV3 and stained with Hoechst. CPE was evaluated by brightfield and fluorescence microscopy. Arrows indicate dense nuclear bodies in Ac-SAV3-infected insect cells.

A recombinant baculovirus was generated (Ac-SAV3) to express the complete SAV3 structural cassette C-E3-E2-6K-E1 (Fig. 1A). The complete coding sequence of the structural cassette was cloned downstream of the polyhedrin promoter of an adapted AcMNPV backbone. Insect cells were infected with a MOI of 10 TCID_50_/cell and were incubated for 72 h. Protein expression in the cell fraction was analyzed by CBB and WB using α-E1 and α-E2 mabs. Total protein-staining by CBB showed high levels of expression based upon the abundant band of approximately 35 kDa, which closely matches the predicted size of the SAV capsid protein (Fig. 1B, left). Western analysis using α-E1 and α-E2 mabs yielded bands of ∼50 kDa and ∼55 kDa, respectively (Fig. 1B, center). These sizes correspond to the predicted molecular mass of E1 (49.2 kDa) and E3E2 (54.8 kDa), suggesting that expression of the SAV3 structural cassette results in an E3E2 intermediate that is not further processed by host furin.

**Figure 2:**
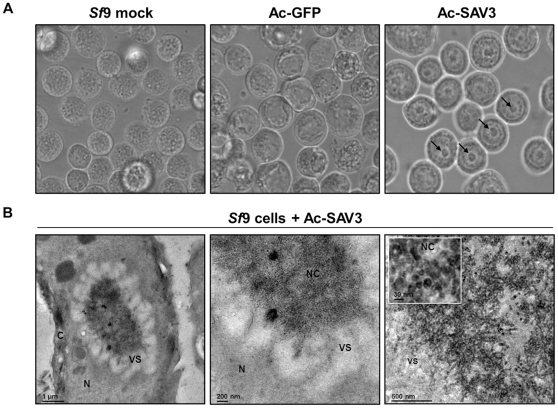
Nuclear localization and assembly of SAV3-nucleocapsids. *Sf*9 infection with Ac-SAV3 and Ac-GFP was analyzed by A) light-microscopy at 2 d.p.i. B) Ac-SAV3-infected cells were fixed and embedded in gelatin and ultrathin coupes were analyzed by TEM, with C, N, VS and NC indicating cytoplasm, nucleus, virogenic stroma and SAV nucleocapsids, respectively.

To analyze if SAV glycoproteins were glycosylated (Fig. 1C), expression products were treated with PNGase F, which enzymatically removes carbohydrate residues from proteins. SAV3-E2 is predicted to be N-glycosylated at N318 (Fig. 1A) [17], [45]. As expected, PNGase F treatment reduced the size of E3E2 with a few kDa (Fig. 1C). The shift in molecular mass of the SAV3-E3E2 fraction after PNGase F treatment suggests that SAV3-E2 is indeed N-glycosylated, when expressed in *Sf*9 insect cell by recombinant baculoviruses. The absence of non-glycosylated protein fraction in the untreated sample indicates that SAV3-E2 is very efficiently glycosylated in insect cells, despite the fact that E3 is not released from E3E2 by furin cleavage. Infected cells displayed baculovirus-specific cytopathic effect (CPE) including decreased cell growth, enlarged nuclei and cell monolayer detachment. However, *Sf*9 cells infected with Ac-SAV3 showed additional CPE. Cell membranes fused between closely neighboring infected cells resulting in polykaryons or syncytia (Fig. 1D). This syncytia formation was most likely induced by the fusogenic activity of SAV-E1, since alphavirus E1 regulates fusion during endocytosis in wildtype infections [17]. In addition to the enlarged nuclei and syncytia, infected *Sf*9 cells contained dense nuclear bodies that were only detectable in cells expressing the SAV structural cassette (Fig. 1D and Fig. 2A, indicated with arrows) Nuclear staining using Hoechst suggested that the nuclear bodies contained nucleic acids (Fig. 1D).

To further investigate the nature of these nuclear bodies, infected cells were fixed and embedded in gelatin after which the cells were analyzed by transmission electron microscopy (TEM) (Fig. 2B). All infected cells developed the dense structures which appeared to consist of aggregated, smaller spherical structures, surrounded by a halo of viral stroma. Based on morphology and size (∼30 nm) and the fact that SAV3-capsid contains a nuclear localization signal [46], the spherical structures corresponds most likely to assembled SAV nucleocapsids.

### Temperature-dependent processing of SAV3 structural proteins

**Figure 3:**
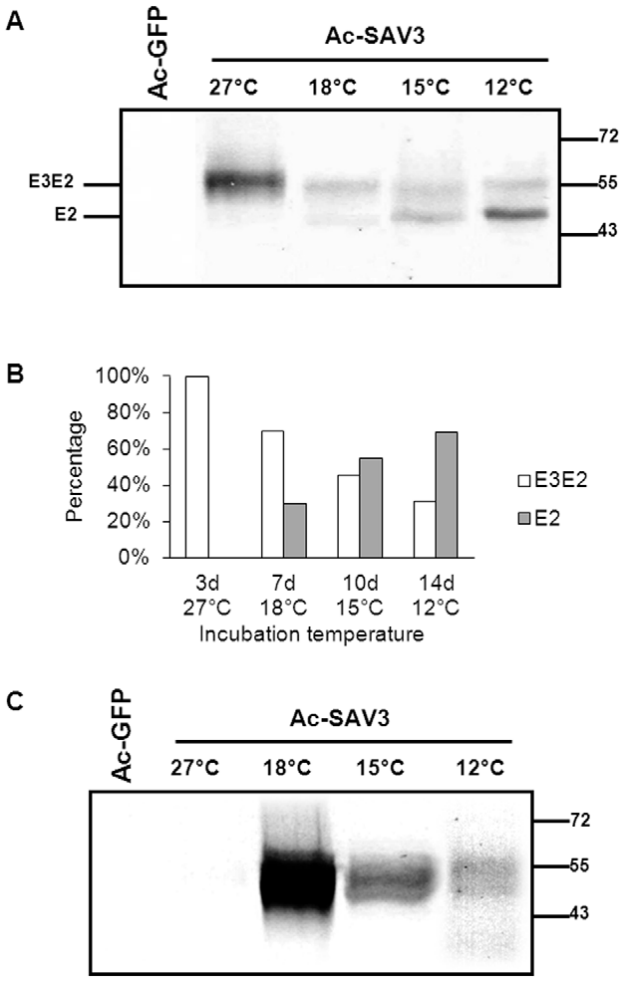
Temperature-dependent processing and secretion of SAV-E2. A) *Sf*9 cells were infected with Ac-SAV3 with an MOI of 10 at 27°C, 18°C, 15°C and 12°C. SAV-E2 expression in the cell-fraction was analyzed by WB using SAV α-E2 mab (17H23). B) Relative percentages of SAV-E3E2 and E2, indicating more efficient processing of E3E2, with decreasing temperatures. C) Secretion of E2 as a function of the temperature. The medium fraction of infected *Sf*9-cell cultures was PEG-precipitated and analyzed by WB using SAV α-E2 mab.

The analysis of the SAV3 structural cassette, expressed by recombinant baculoviruses at 27°C, showed that SAV3-E2 was efficiently glycosylated, but was not released from its E3E2 precursor by host furin-like proteases. Since SAV is an alphavirus of cold-water fish, the lack of furin-processing in E3E2 might be caused by the significant difference in environmental temperature between wildtype SAV replication (12-15°C) [3] and baculovirus expression (27°C). To investigate this putative temperature-effect on furin-dependent processing, *Sf*9-cells were infected with Ac-SAV3 and incubated at 12°C, 15°C, 18°C and 27°C for 14, 10, 7 and 3 days, respectively. Whole cell lysates were treated with PNGase F and analyzed using WB. Expression at 27°C (Fig. 3A, lane 2) resulted in similar protein patterns as seen before (Fig. 1B). The 55 kDa protein band, corresponding to glycosylated but unprocessed SAV-E3E2, was also detected at all lower expression temperatures (Fig. 3A, lane 3, 4, 5). However, in addition to E3E2, a second protein band of lower molecular mass was found. The smaller polypeptide (48 kDa) matches the predicted molecular mass of processed SAV3-E2 (46.9 kDa). This suggests that furin cleavage of E3E2 is rescued at 18°C, 15°C and 12°C. At all temperatures, PNGase I treatment resulted in an equal downward shift of both bands, suggesting that recombinant E3E2 and E2 were N-glycosylated (data not shown). From the relative intensities of the bands on the WB, it could be concluded that the intensity of the E2 fraction increased at lower temperatures. In conclusion, by decreasing the expression temperature from 27°C to 18°C, 15°C or 12°C, the ratio between E3E2 and fully processed E2 shifts towards the processed fraction (Fig. 3B). These results suggest that furin processing of the SAV3 E3E2 precursor is temperature dependent. Medium fractions of Ac-SAV3 infected Sf9 cells were PEG-precipitated to concentrate protein content and western analysis using α-E2 mabs showed that E2 can be detected in the medium fraction (Fig. 3C), but only when proteins were expressed at 12°C, 15°C or 18°C, but not at 27°C.

### Surface expression of baculovirus expressed SAV3-E2 in *Sf*9 cells

**Figure 4:**
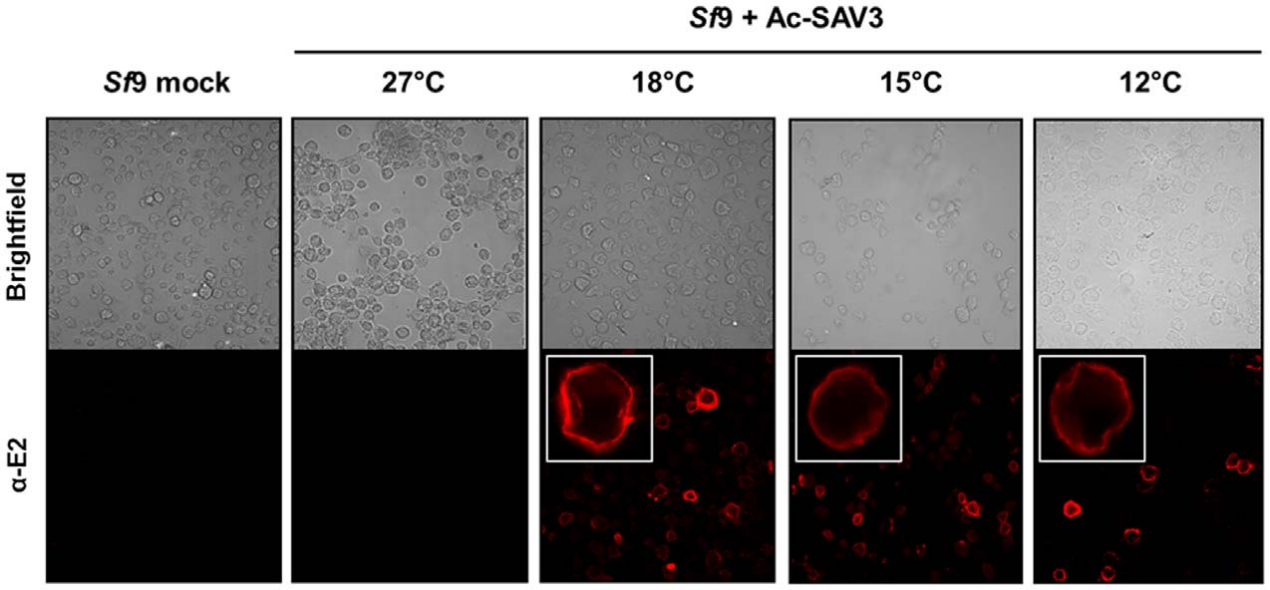
SAV3-E2 detection on the surface of *Sf*9 cells after recombinant baculovirus expression. Cells were infected with Ac-SAV3 at 12°C, 15°C, 18°C and 27°C. Cells were fixed with 4% paraformaldehyde and subjected to immunostaining with α-E2 mabs. Cells were analyzed by confocal microscopy and positive staining indicates the presence of E2 at the surface of infected cells.

To generate progeny viral particles through budding, alphavirus glycoproteins assemble into heterodimers, three of which congregate into trimers, the so-called trimeric spikes. The spikes are formed in the ER and pass through the Golgi apparatus. At the end of the processing pathway, the trimeric spikes are anchored in the plasma membrane by the C-terminal transmembrane domains of E1 and E2 and are exposed on the surface of infected cells [17]. So far, it has been shown that expression of the SAV3 structural cassette by recombinant baculoviruses in insect cells results in the formation of glycosylated E3E2, which, at lower temperatures, is processed to glycosylated E2. This suggests that processing in insect cells resembles the processing as it takes place during wildtype infections in fish cells. In this case, glycoprotein spikes are exposed at the insect cell surface [13]. To analyze whether or not this is true, *Sf*9 cells were infected with Ac-SAV3 at different temperatures. Next, non-permeable cells were subjected to immunofluorescence using α-E2 mabs (Fig. 4). Positive staining indicates that SAV3-E2 is exposed at the surface of the cells. Confocal microscopy revealed that the ring-like structures indicating surface expression could only be observed when Ac-SAV3 was expressed at 12°C, 15°C and 18°C. (Fig. 4). In sharp contrast, surface expression of E2 was not detected at 27°C (Fig. 4), while western analysis showed that E3E2 was expressed at high levels (Fig. 3A). No staining was observed in the mock cells (Fig. 4, left). These data comply with the previous results and demonstrate that the processing of SAV3-E2 is temperature dependent and that E2 can only be detected at the surface of infected cells at lower temperatures.

### SAV3 virus-like particle formation in *Sf*9 cells expressing the SAV3 structural cassette

**Figure 5:**
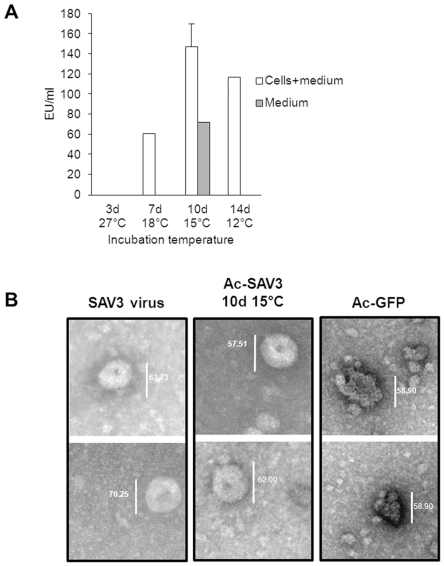
SAV-E2 antigenic mass determination and VLP production. A) SAV-E2 antigenic mass was determined using the SAV-neutralizing mab 17H23 on cell and medium fractions of infected *Sf*9-cells, incubated at 27°C, 18°C, 15°C and 12°C. B) The medium fraction of cells infected with Ac-SAV3 at 15°C was analyzed by TEM to analyze SAV VLP production. Medium fractions of Ac-GFP infected *Sf*9 cells and SAV3 infected Chinook salmon embryo cells were used as control samples.

Processing of the SAV3 structural cassette, expressed by recombinant baculoviruses in insect cells is temperature dependent. Mature E2 is produced at temperatures ranging between 12°C and 18°C, where it is then translocated to the surface of Ac-SAV3 infected cells and subsequently can be detected in the medium fraction. In addition, it was shown that the capsid protein is successfully auto-cleaved from the structural polyprotein (Fig. 1B, left) and E1 is successfully released from the envelope cassette (Fig. 1B, middle). When the structural cassette was expressed at 27°C, E2 retained in the cell fraction in its E3E2 precursor form (Fig. 3A, lane 2). The effect of temperature on SAV maturation is clear, but to asses if temperature has an influence on the antigenicity of recombinant SAV-E2, which carries the major neutralizing epitopes, an immune-capture antigenic mass ELISA using the SAV neutralizing mab (17H23) was performed on total cell culture lysates and medium fraction of Sf9 cells infected with Ac-SAV3 at 27°C, 18°C, 15°C and 12°C (Fig. 5A). The highest antigenic mass was detected in the combined sonicated culture fraction and medium fraction of cells incubated at 15°C. Slightly lower antigenic mass levels were detected at cells incubated at 18°C and 12°C, whereas no antigenic mass was detected at 27°C. Detection of high antigenic mass in the medium fraction of infected cells incubated at 15°C or lower, suggested the formation of SAV VLPs. To investigate whether or not SAV VLPs could be detected, the 15°C medium fraction was evaluated by transmission electron microscopy (TEM) (Fig. 5B). VLP structures – spherical, sometimes donut-shaped, particles of 65–70 nm – that were morphologically similar to SAV3 virus particles (Fig. 5B, left) were found (Fig. 5B, middle), but these were absent in the control medium of Ac-GFP infection (Fig. 5B, right). This shows that SAV VLPs can be produced by expressing the SAV3 structural cassette, using the recombinant baculovirus-insect cell expression system. Moreover, these VLPs can be detected by a SAV-neutralizing mab, suggesting that these VLPs morphologically and antigenically resemble authentic SAV virions.

### SAV3 structural cassette expression and VLP formation by temperature-shift in *Sf*9 insect cells

**Figure 6:**
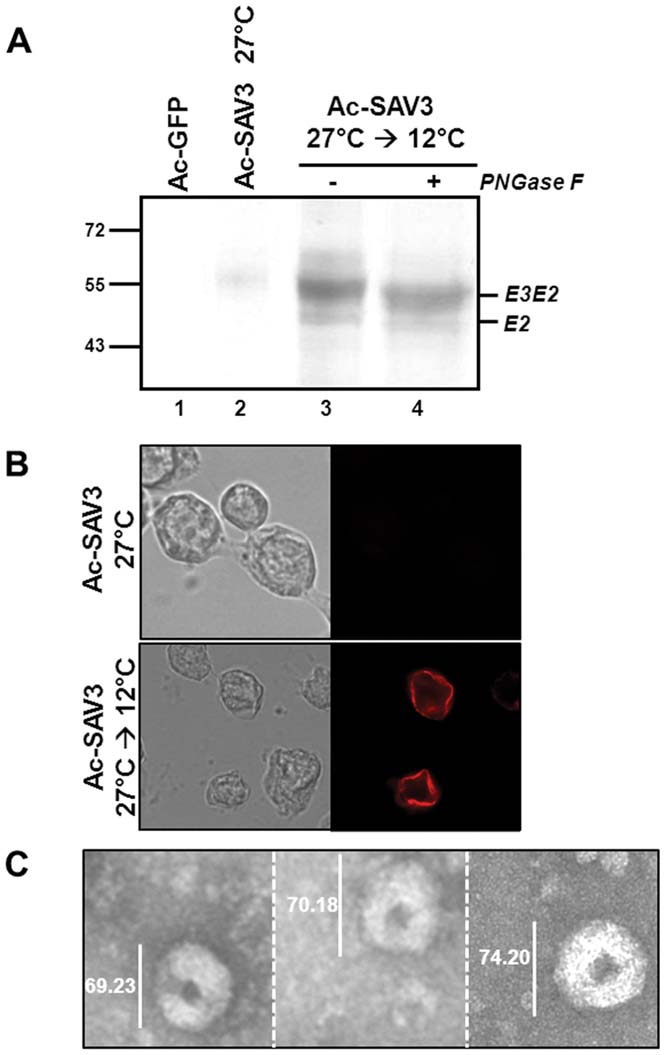
SAV3 structural cassette expression and VLP formation by temperature-shift in *Sf*9 insect cells. *Sf*9 cells were infected with Ac-SAV3, incubated for 2 days at 27°C and subsequently transferred to 12°C for 3 days. A) Cell cultures were treated with/without PNGase F and analyzed by WB using α-E2 mabs. B) Infected cells were treated with 4% paraformaldehyde and subjected to surface immunostaining with α-E2 mabs. C) The medium fraction the infected cell culture was analyzed by TEM for the presence of VLPs.

The results so far have shown that SAV glycoprotein processing and secretion and VLP formation is dependent on the expression temperature. Although SAV-E2 processing appears to be most efficient at 12°C (Fig. 3A), expression at this temperature remains inefficient, due to the low metabolic rate of Sf9 cells and the extensive incubation time of 14 days. To optimize insect cell infection and SAV3 structural polyprotein processing, a temperature-shift experiment was performed. In this experiment, Sf9 cells were first infected with Ac-SAV3 at 27°C for 2 days to allow efficient baculovirus replication, after which the cells were transferred to 12°C for 3 days, to allow expression of properly processed SAV structural proteins. Protein expression, E2 processing, surface localization and VLP formation was analyzed 5 dpi by WB and immunostaining using α-E2 mabs and by TEM (Fig. 6). The temperature-shift resulted in increased processing of SAV3-E2 (Fig. 6A, lane 3), as compared to 27°C expression (Fig. 6A, lane 2), since both E3E2 (∼55 kDa) and mature E2 (∼48 kDa) were detected. PNGase F treatment led to a decrease in molecular mass, showing that both E2 configurations were glycosylated (Fig. 6A, lane 4). Immunostaining on non-permeable Sf9 cells that were infected following the temperature-shift regime showed that E2 was detected on the surface of infected cells (Fig.6B), similar to results found after 12°C incubation (Fig. 4). In contrast, surface staining of the cells infected at 27°C were negative (Fig. 6B). In addition, medium of infected Sf9 cells with the temperature-shift was examined by electron microscopy (Fig. 6C). Characteristic VLP structures were again detected, which were similar to those found after expression at 15°C (Fig. 5B).

**Figure 7:**
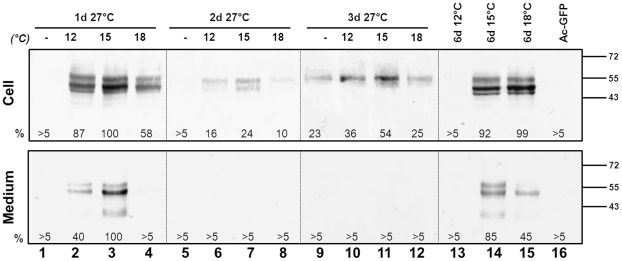
Comparative temperature-shift assay on *Sf*9-cells expressing the SAV3 structural polyprotein by recombinant baculoviruses. Cells were infected with Ac-SAV3 at 27°C for 1, 2 or 3 days. Next, cells were transferred for 3 days to 12°C, 15°C or 18°C. Whole cell lysates and PEG-precipitated medium fractions were analyzed by WB using α-E2 mabs. Protein fraction quantities, relative to 1 day 27°C followed by 3 days 15°C, are indicated.

To further optimize the VLP formation in a temperature-shift regime, *Sf*9 cells were infected with Ac-SAV3 at 27°C and transferred to 12°C, 15°C and 18°C at 1 dpi, 2 dpi and 3 dpi. Cells were subsequently incubated for 3 days at the indicated temperatures. As controls, cells were infected and incubated for a total of 6 days at 12°C, 15°C and 18°C. Both cell and PEG-precipitated medium fractions were analyzed by western analysis using α-E2 mabs (Fig. 7). SAV3-E2 was not detected in both the cell and medium fraction of cells that were infected at 27°C for 1 day (Fig. 7, lane 1). However, when cells were subsequently incubated for three days at lower temperatures, E2 was abundantly detected in both cell and medium fractions (Fig. 7, lane 2-4), indicating a strong increase in processing efficiency due to the temperature shift. Cells that were infected at 27°C for 2 days produced a low amount of E3E2. Although less pronounced than the shift after 1 dpi, the temperature-shift to lower temperatures 2 dpi increased processing and detection of E2 in the cell and medium fractions (Fig. 7, lane 5–8). Here, a shift to 15°C appeared optimal (Fig. 7, lane 7). Infection for 3 days at 27°C, as expected, appeared to be highly disadvantageous for the processing and secretion of E2, since only unprocessed E3E2, but no mature E2 was detected in both cell and medium fractions (Fig. 7, lane 9). It was concluded from this large temperature-shift experiment that infection for 1 day at 27°C followed by expression for 3 days at 15°C was optimal for E2 expression, processing and VLP secretion into the medium. In addition, this temperature-shift regime of in total 4 days significantly shortens infection time as compared to a 6 day infection at 15°C (Fig. 7, lane 14).

## Discussion

SAV infections cause severe economic losses in European aquaculture of Atlantic salmon and rainbow trout. Next to common infection control measures, vaccination is a most effective tool to reduce SAV infections in salmonids. The presently available vaccines are based upon inactivated or attenuated-live vaccine strategies. However, a VLP-subunit approach may serve as an elegant and effective alternative to the safety issues that accompany the present way of vaccine production. The recombinant baculovirus-insect cells expression system has proven to have a high potential in the generation of VLP-subunit vaccines [29], [36]. In this study, SAV VLPs were generated using recombinant baculoviruses and it was shown that the processing of SAV glycoproteins is a temperature-dependent process. VLP formation in insect cells at the normal production temperature of 27°C is not possible.

The SAV3 structural cassette (C-E3-E2-6K-E1) was cloned downstream of the polyhedrin promoter of AcMNPV (Ac-SAV3) and expressed in *Sf*9 insect cells. SDS-PAGE and CBB analysis indicate that the SAV structural (glycol)proteins were expressed in high amounts and that the capsid protein was autocatalytically released from the structural polyprotein, similar as in wildtype alphavirus infection [17], [47]. Alphavirus capsids are multifunctional proteins that are involved in encapsidation of viral genomic RNA, viral budding and in New-world alphaviruses induce host-cell transcription/translational shut-off [17], [48]. Depending on the alphavirus species and the cell type used for expression, capsid localizes in several cellular compartments e.g. cytoplasm, nucleus/nucleoli and mitochondria [46], [48], [49], [50]. Infection of *Sf*9 cells with Ac-SAV3 resulted in the formation of dense nuclear bodies, which appeared to be specific for cells expressing the SAV structural polyprotein. In addition, Hoechst staining showed that nucleic acids co-localized with the dense nuclear bodies and TEM analysis confirmed the *in silico* predicted nuclear localization of capsid [46]. This is the first report that shows the presence of assembled alphavirus nucleocapsids of ∼30 nm in the nucleus of insect cells.

The expression of the SAV-structural cassette in *Sf*9 cells at 27°C clearly prevented complete processing of E2. In this regard, the efficient glycosylation of SAV glycoproteins is remarkable, since our other work showed that CHIKV glycoprotein expression by recombinant baculoviruses resulted only in partial glycosylation [36].

Processing of SAV-E2 by host-furin cleavage was rescued after decreasing the infection temperatures to 18°C, 15°C or 12°C. Immunostaining indicated that only correctly folded proteins were recognized by the α-E2 mab, since at 27°C E3E2 could be detected under denaturing conditions by western analysis, but was not detected by immunostaining in permeabilized cells infected with Ac-SAV3. It has previously been shown that the 17H23 mab recognizes a discontinuous epitope (aa139-306) [43] on SAV3-E2. Incorrect folding of native PE2 might prevent the binding of mab 17H23 to the epitope, while under denaturing conditions, the conformational epitope is restored, thereby allowing antibody recognition by western analysis. Similar epitope-reformation under standard denaturing conditions has previously been described for other viral denaturation-resistant epitopes [51], [52].

The incorrect folding of PE2, at 27°C that prevents binding of mab 17H23 might also render the SAV-PE2 furin-cleavage signal 68RKKR inaccessible to host furin-like proteases. An alternative explanation is that cellular furin is inactive at 27°C, however, this is highly unlikely given that baculovirus F protein is also activated by furin at similar temperatures (26°C–28°C) [53]. The apparent prevention of furin cleavage itself does not fully explain the absence of E3E2 in the medium at 27°C, because furin cleavage is not a prerequisite for alphavirus budding or E3E2 secretion [36], [54], [55]. The deficiency in E3E2 processing and lack of secreted E3E2 at 27°C, is therefore most likely caused by the retention of misfolded SAV-E3E2 in the ER or secretory pathway, in any case upstream of the *trans*-Golgi system where furin-dependent maturation takes place [13]. Misfolded and unfolded proteins usually accumulate in the ER, causing ER stress and thereby disrupting ER functions [56], a phenomenon often observed during overexpression of glycoproteins by recombinant baculoviruses [57]. It will be important to investigate in future experiments which structural change of E3E2 determines its intracellular retention at 27°C.

We show by electron microscopy that recombinant baculovirus expression of the SAV structural cassette in *Sf*9 cells at lower temperatures results in the formation of SAV VLPs. This result was confirmed by western analysis on the medium fraction of infected cell cultures and by sandwich immune-capture ELISA on infected cells and/or medium, in which SAV proteins were only detected at expression temperatures below 27°C. The spherical particles of ∼65 nm in size, morphologically indistinguishable from SAV3 virus particles and other alphavirus VLPs [27], were found exclusively in the medium of Ac-SAV3 infected S*f*9 cells at lower temperatures, but not in the medium of control baculovirus lacking SAV sequences or mock-infected *Sf*9 cells.

Since total production levels of recombinant SAV protein were the highest at 27°C, while lower temperatures were essential for PE2 processing, a hybrid protein production process, with an infection phase at 27°C followed by a production/processing phase at 12°C was developed.

Immunostaining and western analysis showed that recombinant SAV proteins produced following the temperature regime, appeared to be folded and processed correctly, and as expected, SAV VLPs were detected in the medium fraction of Ac-SAV3 infected cells. The temperature-shift production and processing regime was further optimized by varying the production time at 27°C. Both cell and medium fraction analysis showed that a 1 day infection phase at 27°C, followed by a 3 day processing phase at 15°C was optimal for combined protein expression and processing efficiency. In addition, it appeared that the vast majority of VLPs produced at 15°C have mature SAV-E2 incorporated in their envelope. However, PE2 medium detection indicated that, in a small fraction of VLPs, E3 was still associated to the trimeric spikes. This common alphavirus feature does not affect cell receptor recognition by E2 [58]. Thus, we expect VLPs carrying a minor E3E2 fraction still to be sufficiently immunogenic, especially considering that E3 from other alphaviruses harbors protective epitopes [59].

In addition to SAV VLPs, also the insect cells expressing correctly folded SAV structurals may be used in veterinary vaccine formulations. Similar to the widely used baculovirus surface display technique, based upon the expression of foreign peptides/epitopes using a chimeric baculovirus GP64 surface glycoprotein [60], SAV glycoproteins are anchored in the insect cell membrane and are displayed at its surface. However, the baculovirus surface display technique may find limited use in SAV glycoprotein production, since the preceding alphavirus E3 peptide with signal sequence is required for correct E2 folding and the use of heterologous signal peptides has recently been shown not to enhance alphavirus glycoprotein production [36].

The temperature-shift clearly rescues SAV glycoprotein processing, most likely via restoring upstream protein misfolding. A similar temperature-dependent folding phenomenon has previously been described for a temperature sensitive mutant of the vesicular stomatitis virus glycoprotein (VSV-Gmut). VSV-Gmut accumulated in the ER due to misfolding at 40°C, but was refolded when the temperature was decreased to 32°C, enabling VSV-Gmut to enter the secretory pathway into the Golgi-complex [61]. In future studies we would like to investigate the molecular mechanisms of presumed SAV glycoprotein misfolding at high temperatures, but it is unlikely that this will lead to a more efficient VLP production process at 27°C in the short term.

We clearly show that the initial incubation period for 1 day at 27°C following inoculation with baculovirus is of high importance for efficient VLP production. Nonetheless, extension of the 27°C baculovirus infection phase, negatively influenced SAV-PE2 processing and inhibited the formation of SAV VLPs. The strong CPE associated with baculovirus infection that usually occurs 2–3 dpi is most likely disabling cells to rescue SAV glycoprotein folding and processing after the shift to lower temperatures. However, a one day 27°C infection phase is highly beneficial and combined with a three day production processing phase at 15°C significantly shortens the time it takes to produce similar SAV E2 proteins levels as a six day production period at 15°C. This embodies a major advantage for large scale industrial antigen production in insect cell-bioreactors.

This study provides clear evidence that recombinant SAV VLPs can be produced in insect cells using baculovirus expression but also that SAV glycoprotein processing and folding is strictly temperature dependent and a critical determinant of VLP production. The proposed temperature-shift regime not only optimizes SAV VLP production in insect cells, but also provides a general principle for other vaccine candidates of cold-blooded infectious agents in insect cell systems. We aim to address the immunogenicity of our SAV VLPs in a follow up study involving a vaccination trial.
